# Effects of Robot-Assisted Activity Using a Communication Robot on Neurological Activity in Older Adults with and without Cognitive Decline

**DOI:** 10.3390/jcm12144818

**Published:** 2023-07-21

**Authors:** Akio Goda, Takaki Shimura, Shin Murata, Takayuki Kodama, Hideki Nakano, Hironori Ohsugi

**Affiliations:** 1 Department of Physical Therapy, Faculty of Health and Medical Sciences, Hokuriku University, 1-1 Taiyogaoka, Kanazawa 920-1180, Japan; 2 BME Research Laboratory, Sosei Ltd., Hamamatsu 432-8002, Japan; 3 Department of Physical Therapy, Faculty of Health Sciences, Kyoto Tachibana University, Kyoto 607-8175, Japan; 4 Department of Physical Therapy, Faculty of Social Work Studies, Josai International University, Togane 283-8555, Japan

**Keywords:** robot-assisted activity, communication robot, elderly, BPSD, EEG, sLORETA

## Abstract

Robot-assisted activity (RAA) using a communication robot (RAA-CR) has been proposed as a tool for alleviating behavioral and psychological symptoms accompanying dementia (BPSD) in patients with cognitive decline. This study aimed to clarify the effects of differences in cognitive function among older adults on changes in active brain areas induced by RAA-CR. Twenty-nine older adults were divided into a cognitive decline group (*n* = 11) and a control group (*n* = 18). The participants individually received a 5-minute RAA session, and their resting EEG activity was measured before and after the session. Brain spatial analysis was performed on recorded EEG data using standardized low-resolution brain electromagnetic tomography. In addition, statistical comparisons of neural activity in the brain were made before and after RAA-CR and between the cognitively impaired and control groups. These results suggest that RAA-CR stimulates neural activity in the region centered on the posterior cingulate gyrus and precuneus in cognitively healthy older adults but does not significantly alter brain neural activity in cognitively impaired older adults. Therefore, modifications to the implementation methods may be necessary to effectively implement RAA-CR in cognitively impaired individuals.

## 1. Introduction

The global surge in cognitive impairment among older adults is attributed to rapidly aging demographics [1]. The range of behavioral and psychological symptoms accompanying dementia (BPSD) [2], which includes agitation, deviant motor actions, anxiety, elation, irritability, depression, apathy, disinhibition, delusions, hallucinations, and changes in sleep and appetite, presents formidable obstacles to caregiving. It is exceedingly challenging for individuals afflicted with BPSD to maintain a home-based lifestyle because of these manifestations [3]. Moreover, a strong correlation has been reported between BPSD and heightened caregiver stress [4]. Hence, implementation of intervention strategies to control the symptoms of BPSD is critical, as it not only empowers older adults to cope with such symptoms to sustain home living but also alleviates the strain on caregivers.

Two distinct forms of treatment exist for BPSD: pharmacological interventions [5] and non-pharmacological approaches [6]. Although both categories have demonstrated efficacy in mitigating problematic behaviors, non-pharmacological therapies have emerged as the preferred modality for managing BPSD owing to the reduced incidence of associated adverse effects.

In recent years, a multitude of nonpharmacological therapeutic techniques have been developed and implemented, including robot-assisted activity (RAA). RAA is a non-pharmacological therapy that enhances the well-being of older individuals through human-robotic interactions developed by Shibata as an alternative to animal-assisted therapy [7,8]. Previous studies involving older adults with dementia have reported beneficial effects of RAA interventions, including reduced agitation [9,10], improved mood state [10], and apathy [11]. RAA is not associated with the risks of animal allergies, infections, bites, and wounds that are seen in animal-assisted therapies. RAA involves different types of robots (pet type, recreational type, communication type, et al.) and has gained interest as a potential tool for alleviating BPSD symptoms caused by social isolation and the desire for social contact [12,13]. Therefore, communication robots used in RAA [14,15,16,17,18] are of interest as tools for providing social interaction and alleviating BPSD. 

RAA’s suppressive impacts on BPSD are primarily quantified using neuropsychological metrics [9,10,11]. However, this approach falls short as it neglects neurophysiological contributions, which are crucial given that the onset of cognitive impairment is a consequence of both functional alterations and organic transformations in the brain. However, the neurophysiological implications of RAA interventions in BPSD remain undetermined [19,20], which is a major challenge in examining the effectiveness of RAA. We previously investigated the neurophysiological effects of the RAA using a communication robot (RAA-CR) in older adults [21,22]. However, we were only able to examine the effects of RAA-CR on neural activity in brain regions referenced by the subject’s specific electrodes, and we were unable to determine the changes that occurred in all brain regions using whole-head analysis. In addition, the results of studies using focal EEG and salivary cortisol [22] suggest that the effects of RAA-CR in older adults differ depending on the presence or absence of cognitive decline.

In this study, we examined the effects of RAA-CR on brain activity in older adults with and without cognitive decline using standardized low-resolution brain electromagnetic tomography (sLORETA) analysis [23], a three-dimensional imaging filter that estimates the spatial distribution of EEG sources. This study aimed to clarify the effects of RAA-CR on active brain areas in older adults with varying cognitive functions using sLORETA analysis. Understanding these effects may improve the neurophysiological effectiveness of performing RAA-CR.

## 2. Materials and Methods

### 2.1. Participants

Measurements were performed at two day-service centers in Japan, and the staff at these centers recruited participants from among the visitors to the day-service centers. We initially included 43 community-dwelling older adults ≥ 65 years of age who visited the two day-service centers on the days that measurements were performed for this study (a total of 9 days) and who required nursing care because of a decline in physical and mental functions (possibility of mild cognitive decline) due to aging or other reasons. We excluded visitors (1) with known orthopedic or nervous system diseases, including motor deficits and sensory disorders; (2) with severe cognitive decline because of which they could not undergo a cognitive function test; and (3) who were considered unsuitable because of factors such as their personality by the staff of the day-service centers who knew the visitors well. Finally, 29 older adults [12 males, 17 females; mean age: 79.6 ± 7.0 years; mean body mass index (BMI): 23.44 ± 3.23 kg/m^2^] were included in the study. None of the participants had previously performed RAA-CR. Written informed consent was obtained from all participants prior to their participation in accordance with the Declaration of Helsinki. The participants’ general cognitive function was measured prior to the intervention using the Mini-Mental State Examination (MMSE) [24]. Based on their MMSE scores, the participants were divided into the cognitive decline group (MMSE score < 24) and the control group (MMSE score ≥ 24), following a cut-off threshold widely used to detect cognitive decline [25]. This study was approved by the Ethics Review Board of Kyoto Tachibana University (18–56).

### 2.2. Study Intervention

Each participant individually received a 5-minute RAA session while sitting on a chair with a backrest, in line with the protocol defined in a previous study [22]. The RAA was performed using Chapit, a communication robot that has the appearance of a stuffed toy and is equipped with a speech-recognition system (RayTron Inc., Osaka, Japan). When a communication robot is used in a clinical setting such as a day-service center, it is expected to be used in a recreational setting where many people are conversing at the same time. When it is used in such an environment, it must be able to distinguish the user’s voice from the surrounding noises (e.g., other people’s voices) and accurately identify the content of the conversation. Chapit’s high speech recognition capability in noisy environments makes it highly adaptable to clinical settings such as daycare services [26]. Chapit recognizes a predetermined phrase and responds using voice reproduction software that mimics a 5-year-old boy. Additionally, Chapits can perform recreational activities such as prefecture quizzes (regional or culturally based quizzes in Japan), arithmetic games, memory games, and singing.

Participants were given Chapit and a paper containing a list of phrases (e.g., “good morning”, “What time is it?” “Please say something interesting”, etc.). The researcher then presented a brief introductory script.

“This is Chapit. Chapit is a communication robot. Chapit will respond when you talk with Chapit. Let us talk with Chapit using words from the phrase list.”

### 2.3. Outcome Measures

EEG measurements are indicators of emotional changes in subjects with high temporal resolution [27]. Resting-state EEG activity was measured for 120 s in the eyes-open condition both before and after the RAA session, while the participants were seated in a chair with a backrest. EEG was performed using the electroencephalograph Discovery 24E (BrainMaster Technologies Inc., Bedford, MA, USA) and an active dry electrode system (Miyuki Giken Co., Ltd., Tokyo, Japan). EEG was recorded using 16 channels (Fp1, Fp2, F3, F4, C3, C4, P3, and P4; O1, O2, T7, T8, Fpz, Fz, Cz, and Pz), based on the international 10–20 system, at a sampling rate of 256 Hz. Reference electrodes were attached to both earlobes, and impedances were kept below 5 kΩ.

### 2.4. Statistical Analyses

The recorded EEG data were processed using the commercial software Brain Vision Analyzer (version 2.0; Brain Products, Munich, Germany). First, the bandpass filter was set to 1–40 Hz and segmented into 2-s epochs. Prior to data analysis, artifact detection was performed visually to exclude eye movements, head movements, muscle movements, and segments of decreased alertness. Next, for imaging analysis, 60 epochs were extracted from the data obtained under each condition, and a frequency analysis was performed. The data within the beta-wave frequency range (21.5 to 30 Hz) were calculated and used for intracerebral spatial analyses using the sLORETA analysis [23], a method that allows a three-dimensional image display of intracerebral neural activity. The values of each voxel in the brain areas displaying neural activity under each condition were calculated as the neural activity (current source density; μA/mm2*10^−3^) and identified in terms of Brodmann’s area and Montreal Neurological Institute coordinates [28]. By averaging data using the sLORETA Averager software, neural activity was calculated as the global field power value [23] in the two groups under resting-state EEG both before and after RAA. In addition, intracerebral neural activities were compared within the cognitive decline and control groups and between the cognitive decline and control groups at each time point (before and after RAA) using the statistics in sLORETA statistical non-parametric maps [29]. In this analysis, neural areas showing significant differences in activity (with a statistical significance level of 5%) were colored, calculated, and drawn. The significance threshold was based on a permutation test with 5000 permutations using log-transformed LORETA values with subject-wise normalization (based on the assumption that differences in intracerebral neural activity produced by the RAA between the cognitive decline and control groups may exist).

## 3. Results

All participants attended the RAA sessions and completed each 5-minute session. Before RAA, there were no significant differences between the two groups in terms of participant sex percentage, age, and BMI (all *p*-values > 0.05; Table 1), except for the MMSE scores (*p* < 0.01).

**Figure 1:**
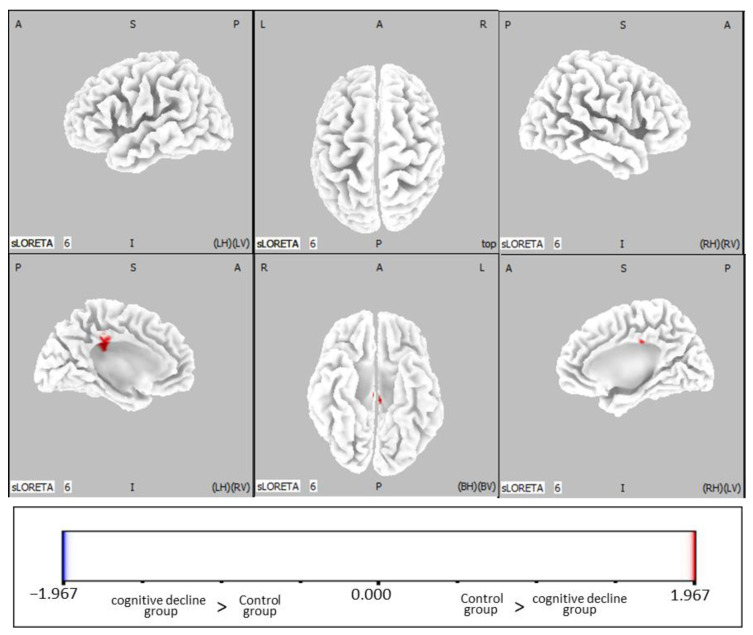
Statistical non-parametric maps (SnPMs) of standardized low-resolution brain electromagnetic tomography (sLORETA) of the beta band comparing the post-rest RAA in the control and cognitive decline groups. Abbreviations: RAA, robot-assisted activity; A, anterior; P, posterior; S, superior; I, inferior; LH, left hemisphere; RH, right hemisphere; BH, both hemispheres; LV, left view; RV, right view; BV, bottom view.

Comparing the cognitive decline group with the control group at each time point (before and after RAA), the beta-band activity in the posterior cingulate gyrus was significantly higher in the control group than in the cognitive decline group after RAA (Figure 1, *p* < 0.05). However, there was no significant group difference in beta band activity before RAA (Table 2).

**Figure 2:**
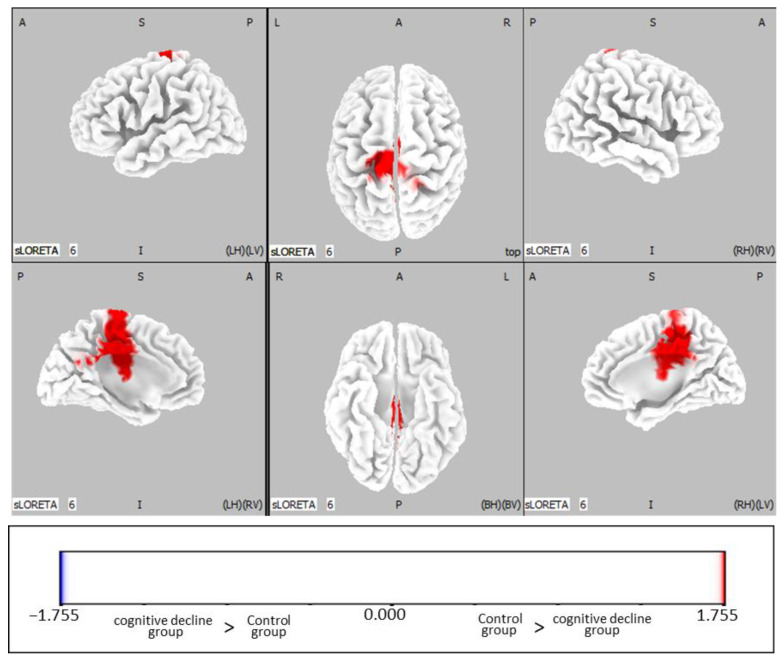
Statistical non-parametric maps (SnPMs) of standardized low-resolution brain electromagnetic tomography (sLORETA) of the beta band comparing pre-rest and post-rest RAA in the control group. Abbreviations: RAA, robot-assisted activity; A, anterior; P, posterior; S, superior; I, inferior; LH, left hemisphere; RH, right hemisphere; BH, both hemispheres; LV, left view; RV, right view; BV, bottom view.

A comparison of the beta-band activity before and after RAA in each group revealed that the beta-band activity was significantly higher in the region centered on the posterior cingulate gyrus (cingulate gyrus, precuneus, paracentral lobule, medial frontal gyrus, posterior cingulate, precentral gyrus, and postcentral gyrus) after RAA in the control group (Figure 2, *p* < 0.05), but there was no significant difference in the cognitive decline group (Table 3).

## 4. Discussion

In this study, we aimed to analyze the effects of RAA-CR on active brain areas in older adults with varying cognitive functions and examine the measures that may be taken to enhance the neurophysiological effects of RAA-CR. Therefore, we investigated the effects of RAA-CR on the brain neural activity of older adults using a three-dimensional imaging filter to estimate the spatial distribution of EEG sources according to whether the subjects were cognitively impaired. The results showed no significant difference between the cognitively impaired and control groups at rest before RAA; however, beta-band activity in the posterior cingulate gyrus was significantly higher in the control group than in the cognitively impaired group at rest after RAA (*p* < 0.05). In addition, a comparison of cranial nerve activity before and after RAA by group showed that the beta-band activity was significantly higher in the region centered on the posterior cingulate gyrus and precuneus after RAA in the control group (*p* < 0.05); however, there was no significant difference in the cognitively impaired group. These results suggest that the effects of RAA on cranial nerve activity in older adults differ owing to differences in the cognitive function levels of the subjects.

There were no significant differences in the resting beta-band cranial nerve activity before the RAA between the cognitively impaired and control groups. It has been reported that the peak frequency and median frequency of the resting EEG of patients with dementia drift toward lower frequencies (e.g., α, θ, and γ) compared to non-demented individuals [30,31,32], but there is no significant difference in beta-band frequency [33,34]; our findings are consistent with the results of these studies.

A comparison of beta-band cranial nerve activity between the cognitively impaired and control groups at rest after RAA showed significantly higher activity in the posterior cingulate gyrus in the control group. The posterior cingulate gyrus has been reported to be a part of the nervous system responsible for self-reflective thinking [35] and is involved in the ability to represent descriptions as mental events during reading [36]. Thus, it is likely that the subjects in the target group reflected on their own experiences through conversation with the robot and recalled mental events through the process of searching for phrases for conversation from the conversation list. It is assumed that the beta-band activity in this part of the brain increased as the neural basis of this process. In contrast, the posterior cingulate gyrus has been reported to cause decreased activity in the early stages of Alzheimer’s disease [37]. Therefore, it is assumed that the cognitive decline group had difficulty activating the neural activity in this region associated with the RAA.

In addition, the brain neural activity in the beta band at rest was compared before and after RAA for each group. In the control group, neural activity in the paracentral lobule, medial frontal gyrus, and pre- and postcentral gyri, which are not directly related to cognitive and emotional processing centers, was significantly higher after RAA than before RAA, in addition to that in the posterior cingulate gyrus and precuneus. The posterior cingulate is linked to internally directed cognition and plays a crucial role in the default mode network, which is active when individuals are engaged in introspection or recalling personal memories [38]. Conversation with the robot may have stimulated personal memory recall or introspective thought, leading to increased activity. The precuneus is associated with self-consciousness, reflective self-awareness, episodic memory, and visuospatial processing [39]. Engagement with the robot could have sparked self-referential thoughts or reflections, leading to increased precuneus activity. Although the paracentral lobule is typically associated with sensorimotor processing, it is also involved in the mental representation of actions, even in the absence of physical movements [40]. Conversing with the robot may have involved mental simulation of actions or responses, which may explain the observed increase in the activity of the paracentral lobule. The medial frontal gyrus is known for its role in social cognition, decision-making, and memory recall [41]. This region might have been engaged because interacting with the robot involved social and cognitive dimensions. The pre- and postcentral gyri are typically associated with actual motor output and sensory input; they can also be engaged through imagination, mental simulation, anticipation, and mirroring of actions [42]. For instance, when older adults speak or intend to speak, they are likely to plan and execute speech articulations, which involve the motor cortex. In addition, when conversing, they may visualize or anticipate actions that are part of the conversation context, even if they are not performing a physical action. For these reasons, there may have been increased beta-band activity in these brain regions in the control group.

These results suggest that RAA-CR activates neural activity in the brain regions associated with reflection, self-consciousness, mental representations of behavior, social cognition, imagination, and prediction in the control group. Individuals with BPSD are thought to have a decreased ability to reflect on themselves, predict the impact of their words and actions on their surroundings, and perceive and choose socially desired words and actions [43]. Therefore, we speculate that the potential of these areas for activation may be a factor that alleviates signs of BPSD.

In contrast to the results of the control group, the cognitively impaired group showed no significant differences in any domain before or after RAA implementation. One possible explanation for this result is the reduced cognitive resources related to conversation, such as memory, comprehension, reasoning ability, and attention function. Cognitively impaired older adults may have reduced cognitive resources and the ability to effectively interact with robots [44]. This may result in reduced engagement in conversation [45] and smaller changes in brain activity. Another possible explanation for this result is the decline in social cognition in the cognitively impaired group. Some cognitive impairments can lead to impaired social cognition [46,47]. In other words, one’s ability to interpret social cues, empathize, and communicate may also be affected. This may result in reduced engagement in interactions with the robot and fewer observable changes in brain activity. Altered brain connectivity may have influenced this result. Cognitive impairment, particularly in conditions such as Alzheimer’s disease, often involves changes in brain connectivity and function [48,49], which may affect the typical patterns of neural responses to cognitive and social stimuli. These events may have contributed to the lack of changes in cranial nerve activity in the cognitively impaired group compared to the control group.

These results suggest that the cognitively impaired group could not understand the conversation with the robot during RAA and could not actively participate in the RAA. Therefore, the following modifications may be necessary to implement the RAA effectively using a communication robot for subjects with cognitive impairment:

- Changing the content of the conversation to be more comprehensible for subjects with cognitive impairment.
- Create a conversation program for the communication robot around content that the cognitively impaired subjects remember well (past enjoyable events, daily routines, etc.).
- Physical contact with the robot (e.g., stroking, hugging) should be possible to provide more stimulation to the subject.
- Increasing the duration or frequency of interventions may improve or increase the effectiveness of RAA-CR.

This study had several limitations. First, the sample size was relatively small and requires careful interpretation. Therefore, future studies with larger sample sizes are warranted. Second, because this study was conducted with older adults who visited day service centers, the results may be relevant only to this population. Further studies in other populations are needed to generalize these findings. Finally, a single 5-min RAA-CR session may not have provided sufficient stimulation to induce changes in neural activity in the cognitively impaired older adults. In the future, longer or more frequent interventions should be conducted to determine the appropriate amount of intervention that may be sufficient to induce cortical neural activity in cognitively impaired older adults.

## 5. Conclusions

In this study, we investigated the effects of RAA using a communication robot on the neural activity of the brain in cognitively healthy and cognitively impaired older adults using sLORETA, a three-dimensional imaging filter that estimates the spatial distribution of EEG sources. The results suggest that RAA using a communication robot stimulates neural activity in the region centered on the posterior cingulate gyrus in cognitively healthy older adults but does not significantly alter brain neural activity in cognitively impaired older adults. Based on the above, to effectively implement RAA using a communication robot for older adults with cognitive decline, it was considered necessary to modify the conversation program of the robot and the implementation format of the RAA so that the participants can understand and actively participate in the conversation content.

## Figures and Tables

**Table 1:** Demographic characteristics of people in the cognitive decline group and the control group.

Variable	Cognitive Decline Group (*n* = 11)	Control Group (*n* = 18)	*p*-Value
Gender (male/female; n)	7/4	5/13	0.12
Age (year)	79.00 (5.46)	79.89 (7.92)	0.75
BMI (kg/m^2^)	21.3 (3.36)	23.40 (3.32)	0.16
MMSE (Score)	17.82 (3.79)	27.53 (2.37)	<0.01

Abbreviations: BMI, body mass index; MMSE, Mini-Mental State Examination.

**Table 2:** Brain regions showing significantly higher activation in the beta band in the control group than in the cognitive decline group after RAA.

		MNI Coordinates	
Brain Region	BA	(x, y, z)	T-Value
Cingulate Gyrus	23 ^1^, 24 ^1^, 31 ^1^	−10, −30, 40	−2.05

^1^ *p* < 0.01; Abbreviations: BA, Brodmann’s area; MNI, Montreal Neurological Institute; RAA, robot-assisted activity.

**Table 3:** Brain regions showing significantly higher activation in the beta band after RAA than before RAA in the control group.

		MNI Coordinates	
Brain Region	BA	(x, y, z)	T-Value
Cingulate gyrus	23 ^1^, 24 ^1^, 31 ^1^	−5, −25, 50	−1.85
Precuneus	7 ^1^, 31 ^1^	−5, −25, 50	−1.85
Paracentral lobule	4 ^1^, 5 ^1^, 6 ^1^	−5, −25, 55	−1.84
Medial frontal gyrus	6 ^1^	−5, −25, 55	−1.84
Posterior cingulate	23 ^1^	0, −25, 35	−1.82
Precentral gyrus	4 ^1^	−15, −30, 65	−1.80
Postcentral gyrus	3 ^1^	−20, −30, 70	−1.79

^1^ *p* < 0.05; Abbreviations: BA, Brodmann’s area; MNI, Montreal Neurological Institute; RAA, robot-assisted activity.

## Data Availability

The datasets analyzed during this study are available from the corresponding author upon reasonable request.
